# Analysis of Factors That Influenced the Mental Health Status of Public Health Workers During the COVID-19 Epidemic Based on Bayesian Networks: A Cross-Sectional Study

**DOI:** 10.3389/fpsyg.2021.755347

**Published:** 2021-12-10

**Authors:** Xin Peng, Yangyang Pu, Xiaoyong Jiang, Qingmei Zheng, Jing Gu, Huan Zhou, Dan Deng

**Affiliations:** ^1^Department of Health Statistics, School of Public Health and Management, Chongqing Medical University, Chongqing, China; ^2^Zigong First People’s Hospital, Zigong, China; ^3^Health Education Institute, Zigong Center for Disease Control and Prevention, Zigong, China; ^4^Chronic Disease Institute, Zigong Center for Disease Control and Prevention, Zigong, China; ^5^School of Public Health, Sun Yat-sen University, Guangzhou, China; ^6^West China School of Public Health, Sichuan University, Chengdu, China

**Keywords:** Bayesian network, COVID-19, anxiety, depression, public health workers

## Abstract

**Background:** Public health workers are essential to responding to the coronavirus disease 2019 (COVID-19) epidemic, but research on anxiety and stress among public health workers during the epidemic is limited. This study aimed to evaluate related factors affecting mental health among public health workers during the epidemic.

**Methods:** Between February 19 and 25, 2020, an online, cross-sectional study was conducted among public health workers in a city in China. Mental health status was assessed using the Chinese versions of the Generalized Anxiety Disorder-7 (GAD-7) scale and Patient Health Questionnaire-9 (PHQ-9), both with a cutoff score of 5. Work-related variables, workloads and sacrifices, and personal perceptions were also assessed.

**Results:** The prevalence of anxiety and depression were 49.2% and 45.7%, respectively, among public health workers. Three risk factors and one protective factor, namely, overcommitment (OR = 1.10∼1.20, *p* < 0.001), perceived troubles at work (OR = 1.14∼1.18, *p* < 0.001), perceived tension (OR = 1.11, *p* < 0.001) and the capability to persist for more than 1 month at the current work intensity (OR = 0.41∼0.42, *p* < 0.001) were found to be independently associated with anxiety and depression in the multivariable logistic regression analyses after propensity score matching. But the Bayesian networks analysis found that the last three factors directly affect anxiety and depression.

**Conclusion:** Psychological responses to COVID-19 were dramatic among public health workers during the severe phase of the outbreak. To minimize the impact of the epidemic, working conditions should be improved, and easily accessible psychological support services should be implemented.

## Introduction

Mental health is an important component of health, and the concept of “no health without mental health” as proposed by the World Health Organization (WHO) has become the consensus view. Mental health problems are currently a prominent challenge worldwide. General estimates suggest that 11–18% of the world’s population meets the criteria for a diagnosable psychiatric disorder at any given time (Wainberg et al., 2017). The drivers of poor mental health include multiple transitions affecting the global population, such as the increasing rates of some social determinants (pandemics, conflict, and displacement) and environmental threats (increased natural disasters associated with climate change) (Yokoyama et al., 2014; Patel et al., 2018). Alarmingly, in crisis situations, such as natural disasters or humanitarian emergencies, the prevalence rate of mental health disorders can reach nearly 22%, which is nearly double the general prevalence (Charlson et al., 2019).

Many studies have suggested that infectious disease epidemics, including severe acute respiratory syndrome (SARS), Middle East respiratory syndrome (MERS), and 2009 novel influenza A (H1N1), are associated with mental health problems among the general population (Cowling et al., 2010; Taha et al., 2014), healthcare workers (HCWs) (Bai et al., 2004; Lee et al., 2018), and patients (Lee et al., 2007; Wang et al., 2011).

At the end of December 2019, an outbreak of coronavirus disease 2019 (COVID-19) occurred in Wuhan, Hubei Province, China (Wang et al., 2020). In only a month, the disease caused by severe acute respiratory syndrome coronavirus 2 (SARS-CoV-2) was declared a public health emergency by the WHO, and it was declared an epidemic in March 2020 (World Health Organization (WHO), 2020a). Due to the severe situation in the early stage of the epidemic, requiring a substantial amount of work in a short time, health systems were strained by the effort to contain the disease, which spread to over 200 countries throughout the world (World Health Organization (WHO), 2020b). In particular, healthcare workers, including public health workers, are the main persons involved in the screening and treatment of this disease, and they remain under great pressure.

The epidemic is still in a severe stage. As of 16 June 2021, there were about two hundred million confirmed cases of COVID-19, including 4,265,903 fatalities, reported to the WHO (World Health Organization (WHO), 2020c). Due to the limited effectiveness and insufficient supply of currently available vaccines, preventive measures, including controlling the sources of infection, impeding transmission, and protecting susceptible populations, are the most effective strategies to contain the spread of the disease (Li et al., 2020). Therefore, public health systems still face significant challenges in countries and regions where local epidemics remain severe. Public health systems vary among countries (Donaldson, 2008); China’s public health system comprises specialized institutions [e.g., the Centers for Disease Control and Prevention (CDC)] as well as primary healthcare institutes (PHIs, e.g., community health centers in urban areas and village clinics in rural areas) (World Health Organization (WHO), 2015). Public health workers are usually affiliated with the CDC or PHIs and are mainly responsible for various prevention and control tasks, including the development of technical instructions, epidemiological investigation of patients and close contacts, surveillance of high-risk populations, collection and examination of specimens, and collection and reporting of data. A high level of overcommitment easily leads to physical and mental stress and exhaustion in healthcare workers (Siegrist, 2008), which, in turn, leads to anxiety and depression. Individuals who are overcommitted wish to be recognized and respected (Siegrist and Li, 2016) and are especially sensitive to work pressure and negative emotions (Du Prel et al., 2018); thus, they face an increased chance of feeling misunderstood and wronged, among other troubles. During the epidemic, public health workers experienced rapid increases in work intensity and work pressure. During periods of intense work, they faced an increased risk of self-perceived work difficulties, which caused physical and mental stress and thus led to anxiety and depression. Overwork has taken a great toll on the physical and mental health of health care workers in particular. Therefore, studying the factors that influence the mental health of public health workers during the COVID-19 epidemic will provide reference data for other countries, protect the health of public health workers and help contain the epidemic.

Studies have shown that epidemics cause serious psychological distress among the general public and healthcare workers (Pappas et al., 2009). Several recent studies have shown that due to overwork, inadequate preparation, and emotional disturbances (such as fear of infection and concerns about family members) attributable to the COVID-19 epidemic, there is a high prevalence of mental health symptoms among medical professionals, including depression, insomnia, anxiety and posttraumatic stress disorder (PTSD) (Cai et al., 2020; Hou et al., 2020; Lai et al., 2020). However, most of the research on the factors influencing mental health has focused on doctors and nurses in hospital settings, rather than public health workers who are mainly affiliated with the CDC and PHIs.

Among the methodologies used to carry out such analyses, the classical methods predominated; such methods include descriptive statistics, multiple linear regressions, hierarchical linear regression, and multivariate logistic regression, among others. Most of these techniques do not allow descriptions of the complex, direct or conditional, and linear or non-linear relationships between the variables considered in the model (García-Herrero et al., 2017). In reality, these factors may not be independent of each other and may have complex network relationships. Bayesian networks (BNs), also called belief networks, combine graph theory and probability theory to explore relationships between variables and are easily interpretable by means of the resulting graph (Larrañaga and Moral, 2011). The model has no strict requirements regarding data distribution; therefore, it can incorporate all the data to reveal the influences of various factors on mental health and the relationships between them. BNs have been widely used in many fields, including social and behavioral science (Aghaabbasi et al., 2020), clinical science (Beaudequin et al., 2020), occupational health (García-Herrero et al., 2017). However, no study to date has used a BN model to investigate the psychological impacts of the COVID-19 epidemic on populations.

Therefore, the aims of the current study were to investigate the mental health status of public health workers during the outbreak and the influences on their mental health by using a BN model and to provide reference data for the development of targeted interventions for this population to improve their mental health during the COVID-19 epidemic. These goals are important for controlling the COVID-19 epidemic and improving public health workers’ long-term health. Moreover, the findings from this investigation will be critical in addressing future outbreaks of emerging infectious diseases.

## Materials and Methods

### Study Design

This cross-sectional study was conducted from February 19 to 25, 2020. Data were collected from a city in China. First, the city was divided into 5 districts and 2 counties according to the administrative plan, and 2 districts and 1 county were randomly selected. Each district/county selected 8 PHIs. The CDC staff of each district/county and the selected PHI staff participated in the survey, which was conducted entirely in China. The link to the questionnaire was distributed among participants (such as CDC workers) through WeChat/QQ working groups.

### Sample Size

According to the purpose of the survey, the World Health Organization (WHO), 1991) recommended formula was used to estimate the total sample size required for this survey:

$$n=\frac{Z^{2}\,P\,\left(1-P\right)}{d^{2}}\times d\,e\,f\,f$$

*P* was estimated based on a previously published study on mental health services during the COVID-19 outbreak in *The Lancet Psychiatry*; the prevalence of anxiety and depression among healthcare workers in Wuhan Province, China, were 44.7% and 50.7%, respectively. Considering that this study was conducted in early February when the epidemic was most severe, the *P*-value was 40% (Liu et al., 2020). *Z* was 1.96 considering a 0.05 type I error. *deff* (design effect) was 2.0. *d* (the allowable absolute error level) was 5%. Considering a response rate of 90%, at least 800 public health workers were required to complete the online survey.

### Participant Recruitment and Online Survey Completion

The participants met the following inclusion criteria: (1) aged 18 years or older; (2) worked at the CDC or PHI of that city during the study period; and (3) participated in COVID-19 control and prevention-related work. All participants were informed of the background, aims, anonymous nature and length (approximately 10–15 min to complete the questionnaire) of the survey. Before the investigation, we conducted rigorous training for the investigators. We limited the training to 1 participant per unique internet protocol (IP) address. Only a completed questionnaire could be submitted. Questionnaires were quality-checked by a researcher to ensure accuracy. A total of 834 participants responded in this study. After strict quality control, 23 participants were excluded because of logical errors. Thus, 811 (97.24%) participants effectively completed the survey. The studies involving human participants were reviewed and approved by the Chongqing Medical University. Completion of the questionnaire indicated informed consent of the participants.

### Measurements

The questionnaire was obtained from the Cultivation Project of the “Three Major” Construction Scientific Research Projects of Sun Yat-sen University in 2020. Data on demographic characteristics [e.g., age, sex, job title, institution, and whether they had children under 6 years old (i.e., the school age)]; COVID-19 control and prevention work-related variables (e.g., type of work, time spent receiving COVID-19 training, knowledge of COVID-19 control and prevention strategies, difficulties in COVID-19 control and prevention); workloads and sacrifices (e.g., number of days with overtime work, number of hours of sleep per day on average, number of days with overnight work, number of working hours per day on average, practices to avoid infecting your family); perceptions related to COVID-19 and work, including whether one persist with more than 1 month or not at their current work intensity; the scores of 2 self-constructed scales (perceived distress at work and perceived tension at work); and the score of the Effort-Reward Imbalance (ERI) scale were collected (Niedhammer et al., 2004).

The primary psychological outcomes included symptoms of anxiety and depression, measured by the 7-item Generalized Anxiety Disorder (GAD-7) scale (Spitzer et al., 2006) and the 9-item Patient Health Questionnaire (PHQ-9) (Wang et al., 2014), respectively. Probable mild anxiety and depression symptoms were defined by a minimum score of 5 points on the respective scales (Kroenke et al., 2001; Spitzer et al., 2006; Liu et al., 2020).

### Definition and Application of Bayesian Networks

Bayesian networks (Koller and Friedman, 2009) consist of directed acyclic graphs (DAGs) and a set of conditional probability distributions (p). Nodes represent random variables, and directed edges represent the direct probability dependence between the corresponding variables *x*_*i*_ and π_*i*_. If there is an edge from π_*i*_ to *x*_*i*_ and the arrow points to *x*_*i*_, then π_*i*_ corresponds to the parents of *x*_*i*_. In this case, the joint probability distribution can be factored in terms of conditioned probabilities:

$$p\,\left(x_{1},x_{2},\ldots ,x_{n}\right)=\prod_{i=1}^{n}p\,\left(x_{i}|\pi_{i}\right)$$

Based on the data, both the graph and probabilities can be automatically constructed (Neapolitan, 2004) following structural learning and parameter learning. First, we need to define a score function to evaluate the degree of fit between the Bayesian network and the training data, and then find the Bayesian network with the best structure based on this score function. In this study, the Akaike information criterion (AIC) algorithm is preferred. This algorithm was proposed by the Japanese statistician Akaike (1974). It is based on the concept of entropy as a standard to measure the quality of a statistical model. The smaller the AIC, the better the model is. The next step was to use maximum likelihood estimation (MLE) (Scutari and Denis, 2014) to learn the parameters to obtain the conditioned probabilities. The goal of MLE is to find a set of parameters θ to maximize the probability of the model producing the observed data as follows:

$${\hat{\theta }}_{M\,L\,E}=\arg m\,a\,x\,\sum_{i=1}^{n}l\,o\,g\,P\,\left(x_{i};\theta \right)$$

This set of parameters is the conditional probability of each node of the Bayesian network.

### Statistical Analysis

Continuous variables are presented as means ± standard deviations. Categorical variables are presented as percentages. Univariate analyses were performed using Student’s *t*-tests for continuous variables and chi-square tests for categorical variables. We used chi-square tests with the Bonferroni correction for multiple comparison. So, the tripartite variables’ the typical significance level of *p*-value ≤ 0.05 was adjusted to *p*-value ≤ 0.05/3 = 0.0167; and the *p*-value of the variable divided into four groups was adjusted to *p*-value ≤ 0.05/4 = 0.0125. Due to the heterogeneity of the data, the demographic variables were not balanced. The measure implemented to control for demographic variables (age, sex, job title, institutions, and having children under 6 years old) was propensity score matching (PSM). The anxiety group and normal group and the depression group and normal group were 1:1 propensity-score matched, and the caliper value was set at 0.02. The data obtained after matching were used in the univariate analyses. The matched data were used for subsequent statistical analyses.

Multivariate forward stepwise logistic regression models were constructed to identify the factors that influenced the anxiety and depression status of the respondents; all the COVID-19-related variables that were found to be significant in the univariate analyses after PSM were entered into the multivariate analysis. Finally, independent factors were identified, and two outcomes (anxiety and depression) were included in the BN analysis. The statistical significance of all analyses was set at *p* < 0.05. IBM SPSS Statistics 20, R 4.0.3 and GeNIe 2.4 Academic were used for data analysis.

## Results

### Univariate Analysis of Potential Factors Related to Anxiety and Depression Before and After Propensity Score Matching

According to the score of GAD-7 and PHQ-9, all participants were divided into the score ≥5 (anxiety or depression) and the score <5 (non-anxiety or non-depression) groups. Of the 811 participants, 399 (49.2%) suffered from anxiety and 371 (45.7%) suffered from depression, as shown in Tables 1, 2. More participants in the anxiety/depression group were younger and had children under 6 years than those in the non-anxiety/non-depression group. In addition, participants in the depression group were likely to be female, CDC worker and intermediate or senior job title than those in the non-depression group.

**TABLE 1:** Demographic characteristics of the anxiety groups before and after PSM (*n*, %).

Demographics	Anxiety (GAD-7 score)					
	Before PSM				After PSM	
		
	≥5 (*n* = 399)	<5 (*n* = 412)	Statistics^a^	≥5 (*n* = 339)	<5 (*n* = 339)	Statistics^a^
**Sex**						
Male	134 (33.58%)	161 (39.08%)	2.643	122 (35.99%)	115 (33.92%)	0.318
Female	265 (66.42%)	251 (60.92%)		217 (64.01%)	224 (66.08%)	
Age	37.96 ± 9.52	41.69 ± 10.40	5.329***	39.37 ± 9.40	39.42 ± 9.71	0.602
**Children under 6 years**						
No	305 (76.44%)	347 (84.22%)	7.788**	277 (81.71%)	276 (81.42%)	0.010
Yes	94 (23.56%)	65 (15.78%)		62 (18.29%)	63 (18.58%)	
**Job title**						
Junior	212 (53.13%)	220 (53.40%)	0.540	179 (52.80%)	180 (53.10%)	0.776
Intermediate/senior	115 (28.82%)	111 (26.94%)		95 (28.02%)	102 (30.09%)	
Other (e.g., volunteer)	72 (18.05%)	81 (19.66%)		65 (19.17%)	57 (16.81%)	
**Institution**						
CDC	142 (35.59%)	122 (29.61%)	3.298	123 (36.28%)	104 (30.68%)	2.391
PHI	257 (64.41%)	290 (70.39%)		216 (63.72%)	235 (69.32%)	

*GAD-7, 7-item Generalized Anxiety Disorder; PSM, propensity score matching; CDC, Centers for Disease Control and Prevention; PHI, primary healthcare institute.*

*^a^χ^2^ analysis was used to test for differences between categorical variables and anxiety groups, and the Student’s t-tests was used to test for differences between continuous variables and anxiety groups.*

**p < 0.01.

***p < 0.001.

**TABLE 2:** Demographic characteristics of the depression groups before and after PSM (*n*, %).

Demographics	Depression (PHQ-9 score)					
	Before PSM				After PSM	
	≥5 (*n* = 371)	<5 (*n* = 440)	Statistics^a^	≥5 (*n* = 305)	<5 (*n* = 305)	Statistics^a^
Sex						
Male	114 (30.72%)	181 (41.14%)	9.422**	109 (35.74%)	103 (33.77%)	0.260
Female	257 (69.27%)	259 (58.86%)		196 (64.26%)	202 (66.23%)	
Age	37.72 ± 9.70	41.66 ± 10.17	5.612***	39.41 ± 9.66	38.67 ± 10.18	−0.918
Children under 6 years						
No	279 (75.20%)	373 (84.77%)	11.697**	249 (81.64%)	244 (80.00%)	0.264
Yes	92 (24.80%)	67 (15.23%)		56 (18.36%)	61 (20.00%)	
Job title						
Junior	197 (53.10%)	235 (53.41%)	6.627*	160 (52.46%)	166 (54.43%)	0.394
Intermediate/senior	116 (31.27%)	110 (25.00%)		93 (30.49%)	86 (28.20%)	
Other (e.g., volunteer)	58 (15.63%)	95 (21.59%)		52 (17.05%)	53 (17.38%)	
Institution						
CDC	151 (40.70%)	113 (25.68%)	20.679***	114 (37.38%)	99 (32.46%)	1.623
PHI	220 (59.30%)	327 (74.32%)		191 (62.62%)	206 (67.54%)	

*PHQ-9, 9-item Patient Health Questionnaire; PSM, propensity score matching; CDC, Centers for Disease Control and Prevention; PHI, primary healthcare institute.*

*^a^χ^2^ analysis was used to test for differences between categorical variables and depression groups, and the Student’s t-tests was used to test for differences between continuous variables and depression groups.*

*p < 0.05.

**p < 0.01.

***p < 0.001.

To further investigate the relationship between COVID-19-related variables and mental health, PSM was used to control for potential covariates. By searching the previous literature and consulting experts, we confirmed that certain demographic variables are risk factors for anxiety and depression (Kisely et al., 2020; Manh Than et al., 2020; Pappa et al., 2020; Xiao et al., 2020). Furthermore, young children are vulnerable to SARS-CoV-2 infection and children under the age of 6 are more likely than older children to have severe and critical cases (Dong et al., 2020); therefore, parents of having children under 6 years old might feel especially burdened by the risk to their families. Rubin and Thomas (1996) suggested that all variables that might be associated with the outcome should be included in PSM. Therefore, we determined that sex, age, children under 6 years, job title, and institution should be included as covariables in the PSM analysis. There were 339 matches between the anxiety group and the normal group and 305 matches between the depression group and the normal group after PSM (Tables 1, 2).

The results of the univariate analyses of potential factors related to anxiety and depression symptoms after PSM are shown in Tables 3, 4. Participants in the anxiety group tended to do both field work and non-field work (81.71% performing both types vs. 18.29% performing only one or the other, *p* = 0.020), have 6–10 difficulties in COVID-19 control and prevention (51.92%, compared to 32.45% with 5 or fewer difficulties; *p* < 0.001, Bonferroni correction), sleep an average of 5–6 h per night (61.06%, compared to 35.40% sleeping more than 6 h per night; *p* < 0.001, Bonferroni correction), work 9–15 h per day (71.39%, compared to 22.42% working 8 or fewer hours per day; *p* < 0.001, Bonferroni correction), be able to persist less than 1 month at their current work intensity (76.99%, compared to 23.01% persist 1 month or more, *p* < 0.001), and perceived the effort-reward balance (57.23%, compared to 42.77% perceiving an imbalance, *p* < 0.001).

**TABLE 3:** Univariate analysis results of variables related to anxiety after PSM (*n*, %).

Variables	Anxiety (GAD-7 score)			
	≥5 (*n* = 339)	<5 (*n* = 339)	Statistics^#^	*P*
**COVID-19 control and prevention work-related variables**				
Type of work				
Field work/non-field work	62 (18.29%)	87 (25.66%)	5.376	**0.020**
Both	277 (81.71%)	252 (74.34%)		
Time spent in COVID-19 training				
≤8 h	70 (20.65%)	71 (20.94%)	3.029	0.220
1–2 days	51 (15.04%)	36 (10.62%)		
>2 days	218 (64.31%)	232 (68.44%)		
Sufficient knowledge of COVID-19 prevention and control measures				
Inadequate/very inadequate	5 (1.47%)	5 (1.47%)	0.509	0.775
Average	88 (25.96%)	80 (23.60%)		
Adequate/relatively adequate	246 (72.57%)	254 (74.93%)		
Number of difficulties in COVID-19 control and prevention				
≤5	110 (32.45%)^a^	192 (56.64%)	46.761	**<0.001**
6–10	176 (51.92%)^b^	129 (38.05%)		
>10	53 (15.63%)^c^	18 (5.31%)		
**Workloads and sacrifices**				
Number of days with overtime work				
≤5	33 (9.73%)	42 (12.39%)	4.316	0.229
6–10	51 (15.04%)	63 (18.58%)		
11–15	43 (12.68%)	48 (14.16%)		
>15	212 (62.54%)	186 (54.87%)		
Average sleep hours per day				
<5 h	12 (3.54%)^a,b^	11 (3.24%)	14.647	**0.001**
5–6 h	207 (61.06%)^b^	159 (46.90%)		
>6 h	120 (35.40%)^a^	169 (49.85%)		
Number of days with overnight work				
0 days	189 (55.75%)	202 (59.59%)	1.259	0.533
1–3 days	94 (27.73%)	82 (24.19%)		
>3 days	56 (16.52%)	55 (16.22%)		
Average work time per day				
≤8 h	76 (22.42%)^a^	127 (37.46%)	19.732	**<0.001**
9–15 h	242 (71.39%)^b^	201 (59.29%)		
>15 h	21 (6.19%)^b^	11 (3.24%)		
Number of practices to avoid infecting your family				
0	40 (11.80%)	39 (11.50%)	2.145	0.342
1–3	244 (71.98%)	258 (76.11%)		
4–7	55 (16.22%)	42 (12.39%)		
**Perceptions**				
How long one can persist with your current intensity of work				
<1 month	261 (76.99%)	184 (54.28%)	38.77	**<0.001**
≥1 month	78 (23.01%)	155 (45.72%)		
Perceived distress at work	13.42 ± 3.26	10.47 ± 3.10	−12.06	**<0.001**
Perceived tension	29.18 ± 5.46	24.07 ± 5.68	−11.94	**<0.001**
Overcommitment	14.78 ± 2.19	13.12 ± 2.39	−9.41	**<0.001**
the effort-reward balance				
No	145 (42.77%)	104 (30.68%)	48.497	**<0.001**
Yes	194 (57.23%)	235 (69.32%)		

*GAD-7, 7-item Generalized Anxiety Disorder; PSM, propensity score matching; COVID-19, coronavirus disease 2019.*

*^#^χ^2^ analysis was used to test for differences between categorical variables and anxiety groups, and the Student’s t-tests was used to test for differences between continuous variables and anxiety groups.*

*Each superscript (a,b,c) letter denotes a subset of variables whose row proportions do not differ significantly from each other at p ≤ 0.05/3 = 0.0167 level. Different subscript letter assignment between groups denotes a significantly different pair of values based on post hoc testing with the Bonferroni correction.*

*Variables with a p-value < 0.05 are in bold.*

**TABLE 4:** Univariate analysis results of variables related to depression after PSM (*n*, %).

Variables	Depression (PHQ-9 score)			
	≥5 (*n* = 305)	<5 (*n* = 305)	Statistics^#^	*P*
**COVID-19 control and prevention work-related variables**				
Type of work				
Field work/non-field work	63 (20.66%)	84 (27.54%)	3.952	**0.047**
Both	242 (79.34%)	221 (72.46%)		
Time spent in COVID-19 training				
≤8 h	61 (20.00%)	66 (21.64%)	2.848	0.241
1–2 days	47 (15.41%)	33 (10.82%)		
>2 days	197 (64.59%)	206 (67.54%)		
Sufficient knowledge of COVID-19 prevention and control measures				
Inadequate/very inadequate	5 (1.64%)	3 (0.98%)	1.163	0.559
Average	76 (24.92%)	68 (22.30%)		
Adequate/relatively adequate	224 (73.44%)	234 (76.72%)		
Number of difficulties in COVID-19 control and prevention				
≤5	105 (34.43%)^a^	173 (56.72%)	33.427	**<0.001**
6–10	156 (51.15%)^b^	113 (37.05%)		
>10	44 (14.43%)^b^	19 (6.23%)		
**Workloads and sacrifices**				
Number of days with overtime work				
≤5	31 (10.16%)^a,b^	43 (14.10%)	8.690	**0.034**
6–10	42 (13.77%)^a^	63 (20.66%)		
11–15	42 (13.77%)^a,b^	37 (12.13%)		
>15	190 (62.30%)^b^	162 (53.11%)		
Average sleep hours per day				
<5 h	11 (3.61%)^a,b^	8 (2.62%)	10.701	**0.005**
5–6 h	181 (59.34%)^b^	144 (47.21%)		
>6 h	113 (37.05%)^a^	153 (50.16%)		
Number of days with overnight work				
0 days	172 (56.39%)	187 (61.31%)	1.610	0.447
1–3 days	81 (26.56%)	74 (24.26%)		
>3 days	52 (17.05%)	44 (14.43%)		
Average work time per day				
≤8 h	82 (26.89%)^a^	115 (37.70%)	8.485	**0.014**
9–15 h	207 (67.87%)^b^	179 (58.69%)		
>15 h	16 (5.25%)^a,b^	11 (3.61%)		
Number of practices to avoid infecting your family				
0	32 (10.49%)	39 (12.79%)	1.499	0.473
1–3	235 (77.05%)	222 (72.79%)		
4–7	38 (12.46%)	44 (14.43%)		
**Perceptions**				
How long one can persist with your current intensity of work				
<1 month	238 (78.03%)	174 (57.05%)	30.629	**<0.001**
≥1 month	67 (21.97%)	131 (42.95%)		
Perceived distress at work	13.27 ± 3.29	10.70 ± 3.15	−9.883	**<0.001**
Perceived tension	28.95 ± 5.67	24.24 ± 5.49	−10.427	**<0.001**
Overcommitment	14.61 ± 2.25	13.30 ± 2.51	−6.773	**<0.001**
The effort-reward balance				
No	130 (42.62%)	206 (67.54%)	38.271	**<0.001**
Yes	175 (57.38%)	99 (32.46%)		

*PHQ-9, 9-item Patient Health Questionnaire; PSM, propensity score matching; COVID-19, coronavirus disease 2019.*

*^#^χ^2^ analysis was used to test for differences between categorical variables and depression groups, and the Student’s t-tests was used to test for differences between continuous variables and depression groups.*

*Each superscript (a,b) letter denotes a subset of variables whose row proportions do not differ significantly from each other (the tripartite variables’ p-value was adjusted to ≤0.05/3 = 0.0167; and the p-value of the variable divided into four groups was adjusted to p-value ≤ 0.05/4 = 0.0125). Different subscript letter assignment between groups denotes a significantly different pair of values based on post hoc testing with the Bonferroni correction.*

*Variables with a p-value < 0.05 are in bold.*

Furthermore, participants in the depression group also tended to do both field work and non-field work (79.34% performing both types vs. 20.66% performing only one or the other, *p* = 0.047), have 6–10 difficulties in COVID-19 control and prevention (51.15%, compared to 34.43% with 5 or fewer difficulties; *p* < 0.001, Bonferroni correction), work overtime more than 15 days (62.30%, compared to 13.77% working overtime 6–10 days; *p* = 0.012 < 0.0125, Bonferroni correction), sleep an average of 5–6 h per night (59.34%, compared to 37.05% sleeping more than 6 h per night; *p* = 0.001, Bonferroni correction) and work 9–15 h per day (67.87%, compared to 26.89% working 8 or fewer hours per day; *p* = 0.006 < 0.0167, Bonferroni correction) per day, be able to persist less than 1 month with current work intensity (78.03%, compared to 21.97% persist 1 month or more, *p* < 0.001), and perceived the effort-reward balance (57.38%, compared to 42.62% perceiving an imbalance, *p* < 0.001). Moreover, participants with depression and anxiety had greater perceived distress, perceived tension, and overcommitment than those without depression or anxiety.

### Multivariate Stepwise Logistic Regression Analysis

The associations between influencing factors and anxiety and depression symptoms among public health workers during the COVID-19 epidemic are shown in Table 5. In the multivariate stepwise logistic regression analysis, persistence for more than 1 month at the current work intensity (OR = 0.41∼0.42, *P* < 0.001) was a significant protective factor against anxiety and depression symptoms, while perceived destress at work (OR = 1.14∼1.18, *P* < 0.001), perceived tension (OR = 1.11, 95% CI: 1.07–1.15, *P* < 0.001), and overcommitment (OR = 1.10∼1.20, *P* < 0.001) were positively associated with anxiety and depression.

**TABLE 5:** Associations between mental health status and background variables (multivariate stepwise logistic regression)^a^.

Variables	Anxiety		Depression	
	OR (95% CI)	*P*	OR (95% CI)	*P*
How long one can persist with your current intensity of work				
<1 month				
≥1 month	0.41 (0.28–0.60)	<0.01	0.42 (0.28–0.62)	<0.01
Perceived distress at work	1.18 (1.11–1.26)	<0.01	1.14 (1.07–1.21)	<0.01
Perceived tension	1.11 (1.07–1.15)	<0.01	1.11 (1.07–1.15)	<0.01
Overcommitment	1.20 (1.10–1.31)	<0.01	1.10 (1.01–1.19)	<0.01

*CI, confidence interval; OR, odds ratio.*

*^a^Variables that were significant in the univariate analyses in Tables 3, 4 entered into the forward stepwise models after adjusting for sociodemographic variables with propensity score matching (sex, age, a child <6 years, job title and institution).*

### Bayesian Network Models

**FIGURE 1:**
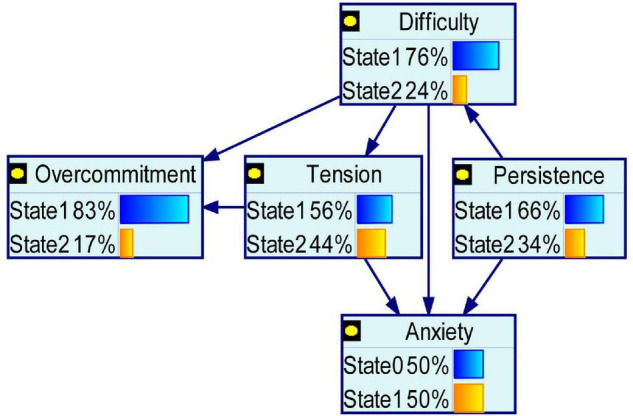
Bayesian network for anxiety. Anxiety and the significant COVID-19-related variables in the multivariate analyses after propensity score matching entered into Bayesian network.

**FIGURE 2:**
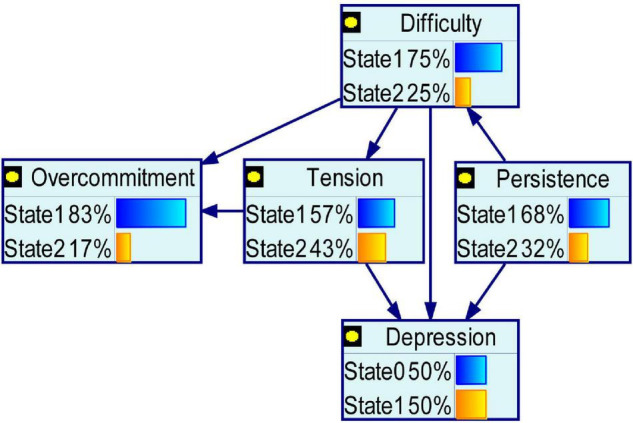
Bayesian network for depression. Depression and the significant COVID-19-related variables in the multivariate analyses after propensity score matching entered into Bayesian network.

According to the result of the multivariate stepwise logistic regression analysis, anxiety and depression had the same 4 influencing factors. Table 6 lists and assigns these 4 influencing factors and outcome variables, which were used in the BN analysis. Figures 1, 2 show BN models of the influencing factors of anxiety and depression symptoms among public health workers during the COVID-19 epidemic, respectively. Persistence, tension, and difficulty directly affected both anxiety and depression.

**TABLE 6:** Variable descriptions and assignments.

Variables	Name	Variable assignment
How long one can persist with your current intensity of work	Persistence	<1 month = 1, ≥1 month = 2
Perceived distress at work	Difficulty	The final score in the lower two-thirds of the range = 1 (low level), the final score in the upper third of the range = 2 (high level)
Perceived tension	Tension	The final score in the lower two-thirds of the range = 1 (low level), the final score in the upper third of the range = 2 (high level)
Overcommitment	Overcommitment	The final score in the lower two-thirds of the range = 1 (low level), the final score in the upper third of the range = 2 (high level)
Anxiety	Anxiety	GAD-7 score <5 = 0, GAD-7 score ≥5 = 1
Depression	Depression	PHQ-9 score <5 = 0, PHQ-9 score ≥5 = 1

Overcommitment was dependent on tension and difficulty. Tension was dependent on difficulty. Difficulty was dependent on persistence. Health status (anxiety and depression) and overcommitment were conditionally independent of tension.

### Conditional Probability Distribution

Tables 7, 8 are conditional probability tables. Because of limited space, we present only a random selection of the conditional probability results.

**TABLE 7:** Conditional probability of anxiety.

Difficulty	Tension	Persistence	Anxiety	
			0	1
1	1	1	0.61	0.39
1	1	2	0.87	0.13
2	1	1	0.31	0.69
2	1	2	0.33	0.67
1	2	1	0.30	0.70
1	2	2	0.49	0.51
2	2	1	0.19	0.81
2	2	2	0.19	0.81

*Variable assignments are shown in Table 6.*

**TABLE 8:** Conditional probability of depression.

Difficulty	Tension	Persistence	Depression	
			0	1
1	1	1	0.59	0.41
1	1	2	0.83	0.17
2	1	1	0.43	0.57
2	1	2	0.63	0.37
1	2	1	0.31	0.69
1	2	2	0.50	0.50
2	2	1	0.19	0.81
2	2	2	0.21	0.79

*Variable assignments are shown in Table 6.*

As shown in Tables 7, 8, P (anxiety = 1| difficulty = 2, tension = 2, persistence = 1) ≈ P (anxiety = 1| difficulty = 2, tension = 2, persistence = 2) = 0.81, P (depression = 1| difficulty = 2, tension = 2, persistence = 1) = 0.81, and P (depression = 1| difficulty = 2, tension = 2, persistence = 2) = 0.79. Through this expression, we can draw the following conclusions. If persistence at the current work intensity occurred for more or less than a month and the scores of perceived troubles at work and perceived tension were high, the probabilities of anxiety and depression were very high (0.81 and 0.79–0.81, respectively).

## Discussion

The present results show that during the COVID-19 epidemic, mental health problems were prominent among public health workers, with 49.2% and 45.7% suffering from anxiety and depression, respectively. These high prevalence rates were similar to the rates reported in a previous study, which found that the prevalence of anxiety and depression among 1,563 medical staff were 44.7% and 50.7%, respectively, using the same measurements and cutoff points (Liu et al., 2020). The mental health of public health workers during the COVID-19 epidemic is of great concern.

Before PSM, there were demographic differences between the groups with different health conditions (anxiety and non-anxiety, depression and non-depression). Research variables related to COVID-19 (e.g., knowledge mastery, perceived troubles at work, perceived tension) may be affected by demographic factors (e.g., age, sex, and professional title). Thus, it is difficult to accurately assess the influence of COVID-19-related variables on mental health status. For reasons involving time and cost, most of the current studies on mental health among various populations during the epidemic are retrospective studies, which cannot be randomized to control for confounding factors to reduce their interference with outcome effect estimates. Therefore, by using PSM to control for confounding factors, the relationships between COVID-19-related variables and the mental health of public health workers could be thoroughly explored.

Univariate analysis after PSM showed that public health workers in the anxiety and depression group were more likely to work 9–15 h and sleep an average of 5–6 h per day than to work 8 or fewer hours and sleep more than 6 h per day. Working long hours potentially reduces the sleep time of public health workers (Marjanovic et al., 2007; Afonso et al., 2017). Those who reported insufficient sleep had adverse mental health effects, similar to the findings in other studies (Simon et al., 2020). During the most severe period of the epidemic in China, a study noted that medical workers had insufficient time to rest (Chen et al., 2020). Public health workers experienced psychological distress were more likely to encounter 6–10 difficulties at work than to encounter five or less, such as poor communication (Naushad et al., 2019) and shortages of medical protective equipment (Guangming Online, 2020; World Health Organization (WHO), 2020d). Moreover, public health workers suffering from anxiety and depression tended to be the effort-reward unbalance. The reason may be that they are not widely understood or respected by the public (Hellewell et al., 2020). During the epidemic, the prevention and control work they perform, such as isolating close contacts (at home or in a designated hotel) and conducting home inspections, is often met with non-cooperation and even opposition. This conflict not only hinders prevention efforts but also has a negative impact on the mental health of public health workers. Time-consuming paperwork and data analyses, especially in emergency situations, may further increase people’s physical and mental health burdens (World Health Organization and International Labour Organisation (WHO and ILO), 2000).

To avoid potentially false associations between the research variables and the dependent variable in the univariate analysis, we performed multivariate logistic regression analysis. The results showed that the impacts of type of work, number of difficulties in COVID-19 control and prevention, average sleep time per day (hours), average work time per day (hours), and work/life balance on mental health were offset. This may be related to the insufficient sample size or the collinearity of independent variables.

Research has shown that overcommitment is a risk factor for declining mental health (Hinsch et al., 2019). If public health workers overexert themselves at work and fail to obtain a reward, their frustration at work increases, potentially leading to anxiety and depression. Public health workers in anxious and depressed states had increased scores for perceived distress; these states were mainly due to their work not being understood, feeling wronged and unfairly treated at work, and regular work pressure. These factors may be related to poor social support, high labor intensity, and high personal costs among public health workers. In the fight against COVID-19, public health workers have been under tremendous pressure and are overworked and exhausted from long hours of intense work, while worrying about themselves and their families contracting the infection (Kang et al., 2020). Persistent internal physical and mental tension may manifest as psychotic symptoms in many public health workers during the epidemic.

Given that a multivariate logistic regression analysis usually considers each influencing factor under the assumption of independence and reveals only the independent factors influencing mental health, a BN model was used to construct a network diagram and conditional probability table to further describe how each factor was interrelated and affected the occurrence of anxiety and depression. The time at current work intensity, perceived troubles at work, and perceived tension were directly related to anxiety and depression, consistent with the results of the multivariate logistic regression analysis.

Mental health issues affect the attention, understanding, and decision-making abilities of public health workers, thereby hindering the fight against COVID-19 and affecting their overall health. Therefore, various interventions should be established to protect the mental health of public health workers. For example, the unit should reasonably schedule working hours, recruit volunteers with medical backgrounds and provide adequate training, thereby distributing the burden of basic public health work and relieving work-related pressure on public health workers. Moreover, social support and public awareness of the importance of public health work should be increased through media campaigns. The difficulties encountered by public health workers should be reduced by the provision of adequate protective equipment, the simplification of reporting data and the introduction of a reasonable and fair system of rewards and punishments. Finally, timely psychological interventions targeting public health workers should be implemented to alleviate stress during the epidemic.

This study has several limitations. First, our study was a cross-sectional study that extracted data from only one point in time. Changes in the mental health status of public health workers in different periods during the epidemic should be investigated. Second, to prevent the potential spread of COVID-19, an online questionnaire was administered. Therefore, there was non-response bias in the study. Third, all the measures relied on self-reported questionnaires, which are dependent on individuals’ subjective accounts and are vulnerable to reporting bias.

## Data Availability Statement

The original contributions presented in the study are included in the article/supplementary material, further inquiries can be directed to the corresponding author/s.

## Ethics Statement

The studies involving human participants were reviewed and approved by the Chongqing Medical University. Written informed consent for participation was not required for this study in accordance with the National Legislation and the Institutional Requirements.

## Author Contributions

JG and HZ were responsible for conceiving and designing the study. XJ and QZ collected the data. XP and YP conducted statistical analyses and drafted the manuscript. XP and DD commented and revised the manuscript. All authors contributed, reviewed, and approved the final manuscript.

## Conflict of Interest

The authors declare that the research was conducted in the absence of any commercial or financial relationships that could be construed as a potential conflict of interest.

## Publisher’s Note

All claims expressed in this article are solely those of the authors and do not necessarily represent those of their affiliated organizations, or those of the publisher, the editors and the reviewers. Any product that may be evaluated in this article, or claim that may be made by its manufacturer, is not guaranteed or endorsed by the publisher.
